# Optimized labeling of bone marrow mesenchymal cells with superparamagnetic iron oxide nanoparticles and *in vivo *visualization by magnetic resonance imaging

**DOI:** 10.1186/1477-3155-9-4

**Published:** 2011-02-09

**Authors:** Jasmin, Ana Luiza M Torres, Henrique MP Nunes, Juliana A Passipieri, Linda A Jelicks, Emerson L Gasparetto, David C Spray, Antonio C Campos de Carvalho, Rosalia Mendez-Otero

**Affiliations:** 1 Instituto de Biofísica Carlos Chagas Filho, Universidade Federal do Rio de Janeiro, Rio de Janeiro, Brazil; 2 Dept. of Neuroscience, Albert Einstein College of Medicine, Bronx, NY, USA; 3 Dept. of Physiology and Biophysics, Albert Einstein College of Medicine, Bronx, NY, USA; 4 Hospital Universitário Clementino Fraga Filho, Universidade Federal do Rio de Janeiro, Rio de Janeiro, Brazil

## Abstract

**Background:**

Stem cell therapy has emerged as a promising addition to traditional treatments for a number of diseases. However, harnessing the therapeutic potential of stem cells requires an understanding of their fate *in vivo*. Non-invasive cell tracking can provide knowledge about mechanisms responsible for functional improvement of host tissue. Superparamagnetic iron oxide nanoparticles (SPIONs) have been used to label and visualize various cell types with magnetic resonance imaging (MRI). In this study we performed experiments designed to investigate the biological properties, including proliferation, viability and differentiation capacity of mesenchymal cells (MSCs) labeled with clinically approved SPIONs.

**Results:**

Rat and mouse MSCs were isolated, cultured, and incubated with dextran-covered SPIONs (ferumoxide) alone or with poly-L-lysine (PLL) or protamine chlorhydrate for 4 or 24 hrs. Labeling efficiency was evaluated by dextran immunocytochemistry and MRI. Cell proliferation and viability were evaluated *in vitro *with Ki67 immunocytochemistry and live/dead assays. Ferumoxide-labeled MSCs could be induced to differentiate to adipocytes, osteocytes and chondrocytes. We analyzed ferumoxide retention in MSCs with or without mitomycin C pretreatment. Approximately 95% MSCs were labeled when incubated with ferumoxide for 4 or 24 hrs in the presence of PLL or protamine, whereas labeling of MSCs incubated with ferumoxide alone was poor. Proliferative capacity was maintained in MSCs incubated with ferumoxide and PLL for 4 hrs, however, after 24 hrs it was reduced. MSCs incubated with ferumoxide and protamine were efficiently visualized by MRI; they maintained proliferation and viability for up to 7 days and remained competent to differentiate. After 21 days MSCs pretreated with mitomycin C still showed a large number of ferumoxide-labeled cells.

**Conclusions:**

The efficient and long lasting uptake and retention of SPIONs by MSCs using a protocol employing ferumoxide and protamine may be applicable to patients, since both ferumoxides and protamine are approved for human use.

## 1. Background

Stem cell transplantation has been explored as a new method to prevent or reverse deleterious effects of several types of tissue injury [1,2]. Mesenchymal stem cells (MSCs) derived from bone marrow have the capacity to differentiate into a number of mesenchymal phenotypes, including adipocytes, osteocytes, chondrocytes and myocytes [3-5]. Moreover, MSCs seem to be immunosuppressive, being able to inhibit T cell proliferation *in vitro *and the function of both naive and memory T cells [6-8] and to suppress the development of monocyte-derived dendritic cells in an *in vitro *system [9]. All these features together with the fact that MSCs can be culture-expanded in large numbers show their great potential to repair or reconstitute a wide array of organs [10].

The success of stem cell therapies in patients requires methods to determine the biodistribution and fate of stem cells without postmortem histology, and the lack of tracking data represents a serious obstacle for the clinical use of cell therapy. Thus, the development of sensitive, non-invasive techniques for tracking cells can provide knowledge about the poorly understood mechanisms responsible for the improvement that has been described in several lesion models [11-13]. Magnetic resonance imaging (MRI) is an excellent tool for high-resolution visualization of the fate of cells after transplantation and for evaluation of cell-based repair, replacement, and therapeutic strategies [13-18]. In addition, this technique has been also used for *in vivo *visualization of endogenous neural stem/progenitor cell migration from subventricular zone in normal and injured animal brains [19-21].

For *in vivo *cell tracking, contrast agents such as superparamagnetic iron oxide nanoparticles (SPIONs) have been successfully used for labeling different mammalian cell types [11,22-25]. Ferumoxides are dextran-coated SPIONs clinically used as an intravenous MRI contrast agent for analyzing liver pathology. The nanoparticles are phagocytosed and accumulate in endosomes of Kupffer cells and reticuloendothelial cells [26]. The particles are biodegradable and incorporated into hemoglobin in red cells within 30 to 40 days or integrated into other metabolic processes [27]. SPIONs tend to aggregate and this has been reduced by coating with dextran or other polymers [28]. Unfortunately, dextran-coated SPIONs do not show sufficient cellular uptake to enable tracking of nonphagocytic cells [29]. However, the cellular uptake of SPIONs by nonphagocytic cells can be facilitated by cationic compounds such as poly-L-lysine (PLL) [29,30] and protamine sulfate [31-33] due to their interaction with the negatively charged cell surface and subsequent endosomal uptake [29,34]. PLL is a synthetic cationic polymer commonly used to enhance cell adhesion to the surface of culture dishes. However, its use has not yet been approved in humans. Protamines are low-molecular-weight arginine-rich proteins (~4000 Da), that are purified from the mature testes of fish. Protamine sulfate is an FDA approved polycationic peptide primarily used as an antidote for heparin anticoagulation [35,36]. It has been administered i.v. to humans at doses of 600-800 mg with minimal toxicity and is well-tolerated by cells *in vitro *[37].

Approval for clinical MRI tracking of labeled stem cells depends on efficient cell labeling that does not exhibit cellular toxic effects and does not elicit side effects. Labeling of MSCs with SPIONs has been studied by a number of groups over the past several years [38-40], but no studies have completely characterized the effects of SPIONs on cell proliferation, survival and differentiation and have concurrently shown retention of these labeling particles for long times.

In this work, we carried out a thorough study on the effect of the SPIONs in MSCs using a refined protocol and we compared two different compounds used to facilitate the incorporation of ferumoxides into the cells, poly-L-lysine and protamine. We analyzed the efficiency of SPIONs to label MSCs during short- or long-term exposure (4 or 24 hrs) both *in vivo *and *in vitro*. Furthermore, we investigated the retention time of SPIONs in the cells for up to 21 days and we analyzed the influence of SPION labeling, using our protocol, on the biological properties (proliferation, viability and differentiation) of MSCs. Our results demonstrate the high potential for long-term SPIONs labeling of MSCs using clinically approved substances.

## 2. Methods

### 2.1. Animals

Experiments were performed on adult male Wistar syngeneic rats (8-12 weeks old) or C57BL/6 mice (8-10 weeks old). All experiments were performed in accordance with the U.S. National Institutes of Health Guide for the Care and Use of Laboratory Animals (NIH Publication No. 80-23), and were approved by the Committee for the Use of Experimental Animals at our institutions (Universidade Federal do Rio de Janeiro and Albert Einstein College of Medicine).

Only mice were used for MRI experiments since our MRI coils are too small to accommodate rats. All other experiments were performed on rats.

### 2.1. Isolation and Cultivation of Rat/Mouse Mesenchymal Cells from Bone Marrow

To obtain bone marrow cells, tibias and femurs were isolated, the epiphyses were removed, the bones were individually inserted in 1 mL automatic pipette polypropylene tips and then put in 15 mL tubes. The bones were centrifuged at 300 × g for 1 min and the pellets suspended in Dulbecco's modified Eagle's medium F-12 (DMEM F-12; Invitrogen Inc., Carlsbad, CA, http://www.invitrogen.com ), supplemented with 10% fetal bovine serum (FBS; Invitrogen Inc.), 2 mM l-glutamine (Invitrogen Inc.), 100 U/mL penicillin (Sigma-Aldrich Co., St. Louis, MO, http://www.sigmaaldrich.com), and 100 μg/mL streptomycin (Sigma-Aldrich Co.). Mononuclear cells were purified by centrifugation in Histopaque 1083 (Sigma-Aldrich Co.) gradient at 400 × g for 30 minutes. After three washes in phosphate-buffered saline (PBS) using centrifugations at 300 × g, the cells were plated in 75 cm^2 ^flasks with supplemented DMEM F-12 and maintained in 5% CO_2 _atmosphere at 37°C. The medium was replaced 48-72 hrs after initial culture to remove nonadherent cells and the adherent cells were grown to confluence before each passage. Medium was replaced three times a week. All experiments were performed on third passage cells.

### 2.2. MSC Labeling

In the present study we used a clinically approved contrast agent, ferumoxide (Feridex IV, Advanced Magnetics Inc., Cambridge, MA, http://www.amagpharma.com). The physical properties of Feridex are as follows: the core iron size is 5 nm, and the hydrodynamic size including the dextran coat is 80-150 nm [38]. To improve the incorporation, a final concentration of 5.0 μg/mL protamine chlorhydrate (Valeant Pharmaceuticals International, São Paulo, SP, Brazil, http://www.valeant.com) or 375 ng/mL PLL (MW = 389.000; Sigma-Aldrich Co.) was used as a facilitator agent. In Brazil, protamine chlorhydrate is clinically approved by The National Health Surveillance Agency (ANVISA) and it has been used as a substitute for protamine sulfate in rescue of heparin anticoagulation.

Protamine chlorhydrate and PLL were separately combined with Feridex in culture medium and gently shaken for 30 minutes at room temperature. The solutions containing Feridex and PLL (FePLL) or protamine (FeProt) were added to adherent cell cultures at a proportion of 1:1 in supplemented DMEM F-12. The final concentration of Feridex in all treated groups was 25 μg/mL. All the groups used in this study are listed in Table 1 except the groups described in section 2.7.

**Table 1:** Experimental groups

Group Name	Transfection Agent	Feridex Exposure Time	Experiment duration
**CTRL**	None	None	24 hours
**CTRL/3d**	None	None	3 days
**CTRL/7d**	None	None	7 days
**PLL 24 h**	Poly-L-lysine	None	24 hours
**Prot 24 h**	Protamine	None	24 hours
**Fe 4 h**	None	4 hours	4 hours
**Fe 24 h**	None	24 hours	24 hours
**FePLL 4 h**	Poly-L-lysine	4 hours	4 hours
**FePLL 24 h**	Poly-L-lysine	24 hours	24 hours
**FeProt 4 h**	Protamine	4 hours	4 hours
**FeProt 24 h**	Protamine	24 hours	24 hours
**FeProt 4 h/24 h**	Protamine	4 hours	24 hours
**FeProt 4 h/3d**	Protamine	4 hours	3 days
**FeProt 4 h/7d**	Protamine	4 hours	7 days

### 2.4. Prussian Blue Staining

After incubation with Feridex, the Prussian blue (PB) method was used to detect iron within the cells in culture. This method induces a reduction of ferric iron to the ferrous state with the formation of a blue ferrocyanide precipitate. For PB staining, MSCs were cultured on glass coverslips coated with 0.2% gelatin, washed twice with warm PBS and fixed for 20 min in 4% paraformaldehyde at 37ºC. After fixation, the cells were washed twice with PBS and incubated with Perls' reagent (20% potassium ferrocyanide and 20% hydrochloric acid) for 20 min at room temperature. Cultures were then washed once in deionized water, dehydrated through graded alcohols and mounted with Entellan (Merck KGaA, Darmstadt, Germany, http://www.merck.de). Samples were observed by light microscopy.

### 2.5. Immunocytochemistry

For immunofluorescence, MSCs were grown and fixed as described above. The cells were washed three times with PBS with 0.1% Triton X-100, incubated with 5% normal goat serum (Sigma-Aldrich) in PBS for 30 min, and then incubated with the primary antibody overnight at 4°C. The MSCs were then incubated with the secondary antibody and mounted with VectaShield (Vector Laboratories Inc., Burlingame, CA, http://www.vectorlabs.com). Immunostaining with anti-dextran (1:1000; mouse monoclonal, Stem Cell Technologies, Vancouver, BC, http://www.stemcell.com) was used to detect Feridex incorporation efficacy by different treatments. The proliferation rate of MSCs labeled with Feridex was evaluated by immunostaining with anti-Ki67 (1:400, rabbit monoclonal, Abcam Inc., Cambridge, MA, http://www.abcam.com).

The secondary antibodies used in this study were: Alexa 488-conjugated goat-anti-mouse IgG (1:400; Invitrogen Inc.) and Cy3-conjugated goat-anti-rabbit IgG (1:1,000; Jackson ImmunoResearch Inc., West Grove, PA, http://www.jacksonimmuno.com). The cell nuclei were counterstained with 0.1% 4',6-diamidino-2-phenylindole (DAPI, Sigma-Aldrich Co.).

### 2.6. Feridex-Labeled MSC Viability/Cytotoxicity

The effect of Feridex on viability of MSCs was determined by Live/dead viability/cytotoxicity kit (Invitrogen Inc.) for up to 7 days after initial exposure. Feridex labeled MSCs were incubated with 1 μM calcein AM (green) and 2 μM ethidium homodimer (EthD-1; red) in PBS for 10 min in 5% CO_2 _atmosphere at 37°C. Thereafter, the glass coverslips containing the MSCs were mounted onto slides, viewed by fluorescent microscopy and the ratio of live/dead (green/red) cells determined.

### 2.7. *In Vitro *Retention of Feridex in MSCs

In this study we analyzed the duration of Feridex retention in MSCs. The groups described in this section are not listed in Table 1.

We analyzed the number of labeled cells up to 21 days of culture. After initial FeProt incubation for 4 hrs, the cells were trypsinized weekly and the number of cells labeled with Feridex was counted at the following time points: 1, 7, 14 and 21 days (this group was called **FeProt 7/7d**).

Because cells in culture proliferate more rapidly than *in vivo*, we used Mitomycin C to reduce proliferation rate. Thus, we incubated the cells with 10 μg/mL Mitomycin C for 3 hrs before FeProt incubation for 4 hrs, and as described for the FeProt 7/7d the cells were trypsinized weekly and the number of labeled cells was counted at the following time points: 1,7, 14 and 21 days (this group was called **FeProt MitC**). We chose Mitomycin C to reduce cellular proliferation since it has been widely used for inhibition of cell proliferation in several cell types.

For both groups described above, we used trypsin during the experiment. To control for the possibility that the trypsinization process might interfere with exocytose of the Feridex, we created a group in which no trypsinization was done. In this group the cells were labeled with FeProt for 4 hrs and maintained in culture for 21 days without trypsinization (this group was called **FeProt 21d**).

### 2.8. Differentiation Studies

To determine if Feridex labeling had adverse effects on MSC differentiation, we performed adipogenic, osteogenic and chondrogenic differentiation assays. MSC cells were incubated with FeProt for 4 hrs before starting the differentiation protocol. Control samples were maintained in supplemented DMEM F-12. In all differentiation studies, the medium was changed every 2-3 days. After differentiation, the cells were fixed as described below.

#### 2.8.1. Adipogenic Differentiation

To verify the adipogenic differentiation potential of labeled MSCs, ~70% confluent cells were cultivated for 3 weeks in DMEM F-12 supplemented with 1 μM dexamethasone, 10 μg/mL insulin, 0.5 μM isobutylemethylxanthine and 200 μM indomethacin. The cells were stained with 0.2% Oil Red O for 30 minutes to reveal the intracellular accumulation of lipid-rich vacuoles. All reagents used in this experiment were from Sigma-Aldrich Co.

#### 2.8.2. Osteogenic Differentiation

Osteogenic differentiation was performed with medium supplemented with 1 μM dexamethasone, 10 mM β-glycerolphosphate, and 0.5 μM ascorbic phosphate for 3 weeks. Calcium deposits were evidenced by 1% Alizarin Red staining for 30 minutes in water. All reagents used in this experiment were from Sigma-Aldrich Co.

#### 2.8.3. Chondrogenic Differentiation

To investigate chondrogenic differentiation potential, labeled MSCs were trypsinized and resuspended in supplemented DMEM-F12 at 1.6 × 10^7 ^cells/mL. To form micromass cultures, the cells were seeded in 7 μl droplets in the center of 24 well plates and cultivated under high humidity conditions. After 2 hrs chondrogenesis media (Invitrogen Inc.) was added to the culture plates and the cells were cultivated for 2 weeks. The micromass formed was embebbed in paraffin, sectioned and the presence of proteoglycans was evaluated by 1% Alcian Blue (Sigma-Aldrich Co.) staining in 3% acetic acid (Sigma-Aldrich Co.) solution for 30 min.

### 2.9. *In Vivo *MRI

To confirm that labeled MSCs could be detected by MRI, mouse cells labeled with FeProt for 4 or 24 hrs were injected (3 × 10^6 ^cells in 30 μL of PBS) through the medial surface in the adductor muscles of the hind leg. In these experiments, we used C57BL/6 mice instead of rats since our MRI coils are too small to accommodate rats. The mouse MSCs were isolated and cultivated as described above (2.1). Labeled MSCs were injected into the muscle 18 hrs before the imaging experiment. To perform the MRI, the animals were anesthetized with isofluorane (2-3% in medical air administered via a nose cone). Mice were positioned head-up in the MRI coil in a 9.4-T GE Omega vertical bore imaging system (Fremont, CA, http://www.gehealthcare.com) equipped with an S50 shielded gradient microimaging accessory and a 40 mm inner diameter-60 mm long ^1^H quadrature birdcage imaging coil. Body temperature was maintained by a water-heating system. Transverse plane images of the mouse at the position of the hind limbs were acquired using a 51-mm field of view with a 128 × 256 matrix size (interpolated to 256 × 256). Routine spin-echo imaging was performed due to limitations of the vertical bore MRI system hardware. Eight contiguous 1 mm thick images were acquired with a 300ms repetition time (TR) and an 18 ms echo time (TE); 4 scans were averaged. Each set of 8 images was acquired in approximately 3 min. In plane, resolution was 200 microns.

We used the ImageJ program (from U.S. National Institutes of Health, Bethesda, MD) to quantify the mean intensity of the dark spots.

After imaging, the mouse was sacrificed and the leg muscles were fixed in 4% paraformaldehyde overnight and incubated with 20% sucrose (Sigma-Aldrich Co.) in PBS for at least 24 hrs in 4ºC for cryopreservation. Thereafter, the tissues were incubated in optimal cutting temperature resin (Sakura Finetek USA Inc., Torrance, CA, http://www.sakuraus.com) and 10 μm frozen sections were collected on microscope slides.

### 2.10. *In Vitro *MRI

After labeling with FeProt for 4 hrs, mouse MSCs were washed three times with PBS, trypsinized, fixed for 20 minutes in 4% paraformaldehyde in 1.5 mL tubes and resuspended in 300 μl of 15% gelatin. Tubes containing 10,000 unlabeled cells/μl and 3,330, 1,660 and 166 labeled cells/μl were positioned in the same coil used for *in vivo *experiments. For this experiment, TR was 1s, TE was 15 ms, and four 0.5 mm thick contiguous images were acquired with the same spin-echo sequence used for *in vivo *imaging. To maximize signal to noise and instrument usage, *in vitro *experiments were set up to run overnight (approximately 9 hours) with 256 scans signal averaged and increased in-plane resolution (100 microns).

The ImageJ program (from U.S. National Institutes of Health) was used to quantify the mean intensity of the acquired images from the 1.5 mL tubes.

### 2.11. Microscope Image Acquisition

The photomicrographs shown in this study were obtained using an Axiovert 200 M microscope (Zeiss, GmbH, Germany, http://www.zeiss.com) equipped with ApoTome system, Axiovert 135 microscope (Zeiss) or a Nikon Eclipse TE300 microscope (Nikon Co., Tokyo, Japan, http://www.nikon.com). Quantifications were performed using AxioVision 4.8 software (Zeiss).

### 2.12. Statistical analysis

At least three independent experiments were performed for each statistical analysis. For quantification of labeling, we acquired random images of each sample using a 20x objective and the number of labeled MSCs with florescent probes was quantified as a percentage of the total number of cells. For ferumoxide incorporation and proliferation rate, we acquired 6 images from different fields per sample and the total number of stained cells was divided by the total number of DAPI-stained cells. For live/dead assays, we acquired 8 images from different fields per sample and we divided the number of green or red cells by the total number of cells (green plus red cells). The number of samples (n) used for quantification is indicated in the figures. Brightfield images were acquired to facilitate the quantification of ferumoxide incorporation by MSCs. It was used to determine the membrane boundaries and to distinguish dextran-positive cells from the background.

Statistical significance was evaluated using one-way ANOVA with Bonferroni's post-test for comparison among multiple groups and t-test for comparison between 2 groups. All calculations were done using GraphPad Prism 5 for Windows (GraphPad Software, San Diego, CA, http://www.graphpad.com).

## 3. Results

### 3.1. MSC Labeling and Proliferation

**Figure 1:**
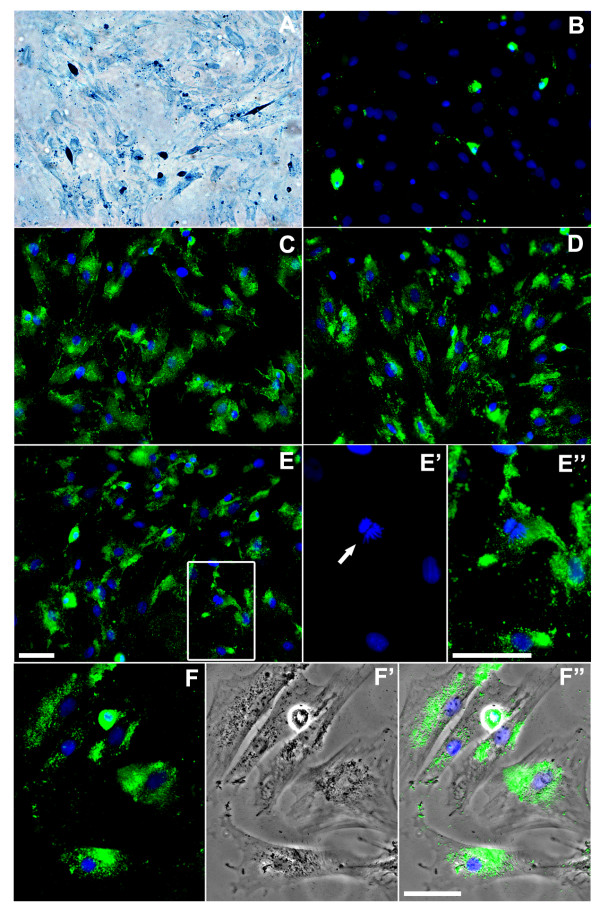
**Representative images demonstrating labeling of MSCs with Feridex in the absence or presence of agents facilitating uptake of the nanoparticles**. (**A-F"**) Presence of Feridex in MSCs was detected by Prussian Blue staining or by dextran immunoreactions. (**A**) Prussian Blue staining in MSCs incubated with FePLL for 24 hrs. Note that virtually all cells display blue intracellular staining. (**B-F"**) Representative images showing dextran immunostaining (green) in MSCs labeled with Feridex and nuclei counterstained with DAPI (blue). (**B**) Cells incubated with Feridex for 24 hrs in the absence of facilitating agents. (**C**-**F"**) MSCs exposed to Feridex in the presence of an agent facilitating incorporation (**C**) FeProt for 4 hrs. (**D**) FePLL for 24 hrs. (**E**) FeProt for 24 hrs. (**E'-E"**) Higher-magnification image of the area indicated by the box in (E) illustrating a Feridex-labeled cell whose nucleus is counterstained with DAPI undergoing mitotic division. (**E'**) Mitotic cell (arrow). (**E"**) Merged image showing DAPI and dextran immunostaining. (**F-F"**) Representative images demonstrating the characteristic perinuclear distribution of Feridex in MSCs after 4 hrs of incubation with FeProt (**F**) DAPI and dextran immunostaining. (**F'**) Brightfield. (**F"**) Merger of the images. Scale bar = 50 μm.

**Figure 2:**
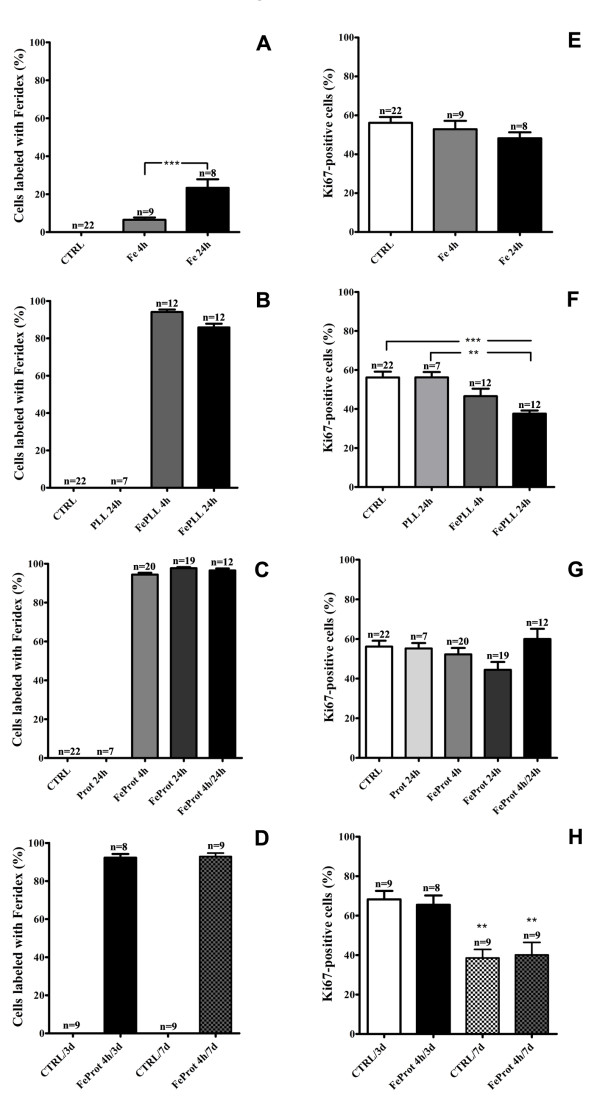
**Quantification of labeling efficacy and proliferation rate of MSCs incubated with Feridex**. (**A-D**) Evaluation of MSC cell labeling by Feridex and/or agents facilitating incorporation for 4 or 24 hrs. (**A**) Cells incubated with Feridex alone for 4 or 24 hrs showed little incorporation. (**B**) Cells exposed to Feridex and PLL showed an efficient incorporation rate. (**C**) Cells exposed to Feridex and protamine showed efficient incorporation of Feridex. (**D**) Quantification of labeling efficacy of MSCs incubated with FeProt complexes for 4 hrs and cultured for up 7 days. The number of cells labeled with Feridex was constant even after 7 days of Feridex incorporation. (**E-H**) Evaluation of proliferative capacity of MSCs exposed to Feridex and/or incorporation facilitator agents for 4 or 24 hrs. Alteration in proliferation rate was observed in MSCs incubated with FePLL complexes for 24 hrs; no change in proliferation was observed in the other groups. (**E**) Cells incubated with Feridex without an incorporation facilitator for 4 or 24 hrs. (**F**) MSCs labeled with Feridex and PLL for 4 or 24 hrs. (**G**) MSCs labeled with Feridex and protamine for 4 or 24 hrs. (**H**) Measurements of proliferative capacity of MSCs incubated with FeProt complexes for 4 hrs and cultured for up 7 days. The proliferation rate was maintained even after 7 days of Feridex incorporation. The "n" indicated on the top of the bars is the number of samples used for the quantification of each group. Error bars represent SEM. **P < 0.01 and ***P < 0.001.

Presence of iron nanoparticles within the cells was confirmed by staining with Prussian Blue (Figure 1A) or anti-dextran antibody (Figure 1B-F"). The Prussian Blue technique was used only to confirm the presence of iron in the cells since we used anti-dextran antibody for quantification of Feridex-positive cells and this antibody only recognizes the dextran coating. It was suggested that dextran coating can undergo degradation when taken up by macrophages [41]. Thus, a limitation in our quantifications is a potential underestimation of the number of labeled MSCs. Efficient Feridex uptake was not observed when the cells were incubated with Feridex without an incorporation facilitator for either 4 or 24 hrs (Figure 2A). However, MSC were efficiently labeled with Feridex when incubated with the facilitating agents, either PLL (Figure 2B) or protamine (Figure 2C). In addition, we did not observe decrease in the number of labeled cells with Feridex at 3 or 7 days after the initial 4 hr incubation with FeProt (Figure 2D).

All groups (except for the group exposed to FePLL for 24 hrs, which showed a lower proliferation rate) incubated with Feridex alone or in combination with incorporation facilitator for 4 or 24 hrs maintained their proliferative capacity when compared to the control group (Figure 2E-G). In addition, no alterations in proliferation rate were observed when we analyzed proliferation for longer periods (3 or 7 days) in the groups exposed to FeProt complexes for 4 hrs comparing with their respective controls (Figure 2H). However, as shown in Figure 2H, the proliferation rate decreased after 7 days of culture when compared with the cultures of 3 days. In the CTRL/7d and FeProt/7d groups, we plated half the amount of cells plated in the CTRL/3d and FeProt/3d groups but the cells approached confluence at 7 days, and the decrease in proliferation rate is probably due to the confluence.

### 3.2. Labeled MSC Viability

**Figure 3:**
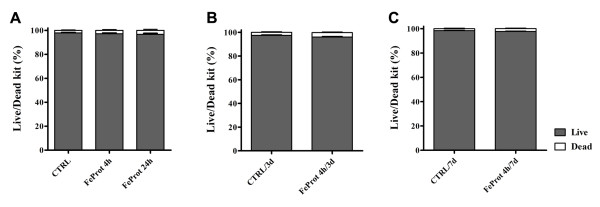
**Evaluation of MSC viability after exposure to FeProt complexes**. (**A-C**) Viability was measured by live/dead assays in live MSCs incubated with FeProt complexes for 4 or 24 hrs. No change in MSCs viability was observed at different time points (**A**) MSCs exposed to FeProt complexes for 4 or 24 hrs. (**B-C**) Viability of MSCs cultured for up 7 days after initial exposure to FeProt complexes for 4 hrs. (**B**) 3 days after initial incubation. (C) 7 days after initial incubation. (N = 9, for each group). Error bars represent SEM.

Live/dead assays were performed in cultures for up to 7 days to evaluate Feridex-labeled MSC viability. Viability assays demonstrated no difference in MSC live/dead ratio after exposing the cells to FeProt for 4 or 24 hrs (Figure 3A) when compared to the control group. Moreover, after longer periods (3 or 7 days) of observation, we did not find alterations in MSC viability after 4 hr exposure to FeProt (Figure 3B-C).

### 3.3. *In Vitro *Retention of Feridex in MSCs

**Figure 4:**
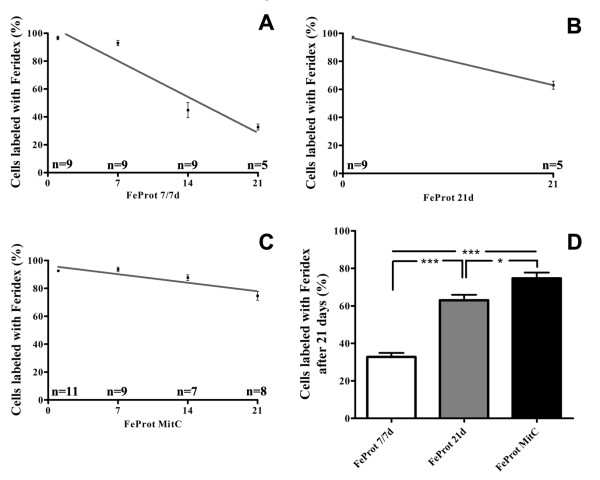
**Quantitative analysis of the duration of Feridex retention in MSCs pretreated or not with mitomycin C**. (**A-D**) MSCs cultured for up 21 days after initial exposure to FeProt complexes for 4 hrs. (**A**) Feridex-labeled cells trypsinized weekly and evaluated after 1, 7, 14 and 21 days of culture. (**B**) The number of MSCs labeled after 1 and 21 days of culture without trypsinization. (**C**) Mitomycin-pretreated cells labeled with Feridex and trypsinized weekly. The number of MSCs labeled was evaluated after 1, 7, 14 and 21 days of culture. (**D**) Comparison of the number of MSCs labeled with Feridex after 21 days of culture in the groups illustrated in (A-C). The percentage of labeled cells was significantly higher in FeProt MitC than in the other groups. The "n" indicated on the top of the bars is the number of samples used for the quantification of each time point. Error bars represent SEM. *P < 0.05 and ***P < 0.001.

We analyzed the duration of Feridex retention in MSCs *in vitro *for up to 21 days after initial incubation with FeProt for 4 hrs. After 21 days of culture we observed a decrease of 66.1%, 32.8% and 19.4% in the number of cells labeled with Feridex in the groups FeProt 7/7d, FeProt 21d and FeProt MitC, respectively (Figure 4A-C). As shown in Figure 4D, the number of MSCs labeled with Feridex was significantly greater in FeProt MitC than in the other groups. In addition, the group FeProt 21d showed a higher number of cells labeled when compared with the group FeProt 7/7d. The fraction of cells labeled with Feridex shown was obtained by immunostaining to dextran, but the presence of iron nanoparticles was confirmed by Prussian Blue staining after 21 days of culture (data not shown).

### 3.4. Differentiation Studies

**Figure 5:**
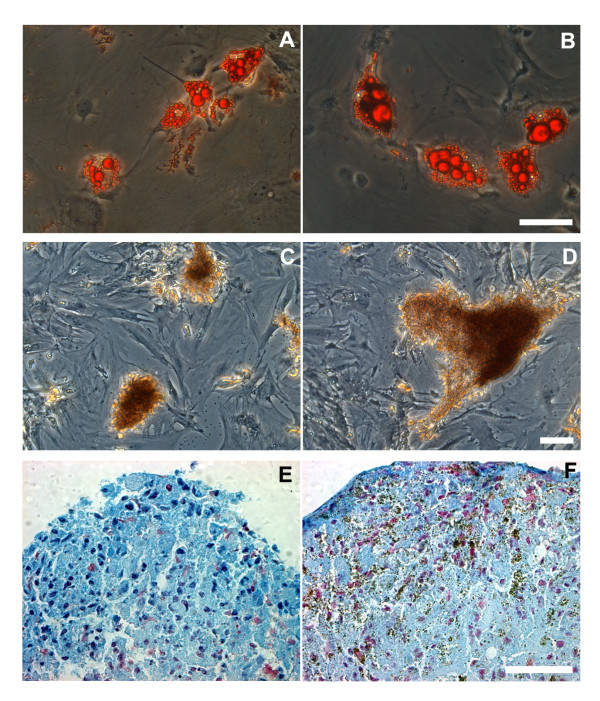
**Analysis of the differentiation potential of MSCs labeled with FeProt complexes for 4 hrs**. (**A-B**) Oil Red O staining indicating adipogenesis in unlabeled or Feridex-labeled cells. (**A**) Unlabeled cells induced toward adipocyte differentiation. (**B**) Labeled MSCs induced toward adipocyte differentiation. (**C-D**) Alizarin Red staining showing osteogenic differentiation in MSCs labeled with Feridex or not. (**C**) Unlabeled cells induced toward osteocyte differentiation. (**D**) Labeled cells induced toward osteocyte differentiation. (**E-F**) Alcian Blue staining showing chondrogenesis in unlabeled or Feridex-labeled MSCs. The nuclei were counterstained with Nuclear Fast Red. (**E**) Unlabeled cells induced to chondrogenic differentiation. (**F**) Labeled cells induced to chodrogenesis. The brown deposits in figure (F) indicate the presence of SPIONs. No apparent alteration in differentiation potential was observed due to Feridex labeling in MSCs. Scale bar = 50 μm.

Differentiation assays were performed *in vitro *in both unlabeled and 4 hrs FeProt labeled MSCs. Staining for intracellular accumulation of lipid-rich vacuoles with Oil Red O revealed that MSCs maintained adipogenic capacity after Feridex incorporation (Figure 5A-B). Also, the osteogenic potential, evidenced by calcium deposits stained with Alizarin Red, was not affected by Feridex labeling (Figure 5C-D). In non-induced cultures we did not observe adipogenic or osteogenic differentiation (data not shown). In addition, unlabeled and Feridex-labeled cells induced toward chondrogenic differentiation formed micromasses that were not observed in non-induced cultures (data not shown). Staining for Alcian Blue revealed the differentiation toward chondrocytes of both unlabeled and labeled cells (Figure 5E-F). Thus, we concluded that FeProt labeling does not impact MSC differentiation into adipocyte, osteocyte or chondrocyte lineages.

### 3.5. *In vivo *and *in vitro *MRI

**Figure 6:**
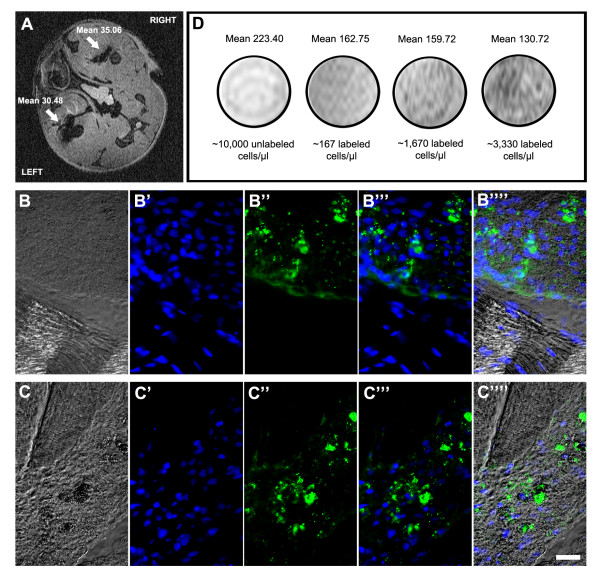
**Detection of Feridex-labeled MSCs by *in vivo *and *in vitro *MRI**. (**A-C**"") Cells labeled with FeProt complexes for 4 or 24 hrs and injected in right or left leg muscles, respectively, were detected by *in vivo *MRI and by dextran immunofluorescence (**A**) Representative image of *in vivo *MRI (transverse plane) showing hypointense (black) spots corresponding to Feridex-labeled cells injected in the leg muscles (white arrows). (**B-B**"") Dextran immunocytochemistry confirming the presence of Feridex-labeled cells in the right leg muscle. (**B**) Phase contrast microscopy. (**B**') Nuclear counterstaining with DAPI. (**B**") Dextran. (**B**"') Merged images showing DAPI (blue) and dextran (green) staining. (**B**"") Merge of images with phase contrast. (**C-C**"") Dextran immunohistochemistry confirming the presence of Feridex-labeled cells in the left leg muscle. (**C**) Phase contrast. (**C**') Nuclear counterstaining with DAPI. (**C**") Dextran. (**C**"') Merged images showing DAPI (blue) and dextran (green) staining. (**C**"") Images merged with phase contrast. Scale bar = 20 μm. (**D**) *In vitro *MRI of unlabeled and FeProt labeled MSCs for 4 hrs. As few as 160 cells/μl could be detected by MRI. The "Mean" values are the mean intensities of the gray values in the range of 0-255.

MSCs labeled with Feridex for either 4 or 24 hrs were detected in mouse tissues by *in vivo *MRI. In the transverse image shown in Figure 6A, the hypointense (dark) spots, indicated by white arrows, show Feridex-labeled cells detected in the mouse legs; the right leg was injected with cells incubated with FeProt for 4 hrs and left leg was injected with cells incubated with FeProt for 24 hrs. There is no apparent difference in the intensity of dark spots in MSCs incubated with FeProt for these different labeling durations. Immunoreaction to dextran confirmed the presence of Feridex-labeled cells in the right and left legs (Figure 6B-C""). In addition, labeled MSCs were detected by *in vitro *MRI. Dark spots were observed in Feridex-labeled cells with density corresponding to number of labeled cells; there was no MRI detection of unlabeled cells, even when the concentration of cells was high (Figure 6D).

## 4. Discussion

Extending knowledge about the effect of SPION incorporation by stem cells is essential for clinical approval of this technique and for its use in tracking stem cells after transplantation. In this study, we evaluated the effect of clinically approved SPIONs in MSCs after short and long-term exposure using the incorporation facilitators PLL and protamine.

The protocol used in this study is different from those used by others. Our choices were based on the following reasoning. The most commonly used concentrations of Feridex are 25, 50 and 100 μg/mL. It was shown that efficient uptake of Feridex (15 to 20 pg of intracytoplasmatic iron/cell) can be achieved using 25 μg/mL Fe and 750 ng/mL PLL in MSC [30,42]. Recently, another group compared four different concentrations of Feridex in human umbilical cord MSCs (5.6, 11.2, 22.4, and 44.8 μg/mL Feridex) and showed that 44.8 μg Fe/mL was toxic for the cells in MTT test [43]. Based on this information we chose to use a low concentration of Feridex (25 μg/mL) in the present study.

In addition, it was shown that the intracellular uptake of iron (pg/cell) is not altered when the ratio of Feridex to protamine varied three fold, from 50:3 FeProt μg/mL to 50:9 FeProt μg/mL [31]. However when a lower concentration of Feridex was used with a lower concentration of protamine (25:0.75 FeProt μg/mL), efficient labeling was not obtained [32]. Therefore, our choice of 25:5 FeProt μg/mL ratio was based on these published observations - a Feridex concentration lower than the reported toxic concentration for cord MSC and a protamine concentration between 3 and 9 μg/mL to test the safety and the efficacy of MSC labeling.

Various concentrations of PLL have been used with 25 μg/mL of Feridex, e.g., 375 ng/mL [44] 750 ng/mL [30,45] and 1500 ng/mL [46]. Since some authors have shown that the FePLL complexes can form toxic aggregates which are not incorporated by the cells [39], we chose a low, but efficient, concentration of PLL.

The MSCs were efficiently labeled with ferumoxide when combined with either facilitating agent, independent of whether the exposure time was short (4 hrs) or long-term (24 hrs). These data corroborate a recent study that showed that the amount of intracellular SPIONs in cells exposed to FeProt for 4, 24 and 48 hours did not change whereas different concentrations of FeProt interfered with the amount of intracellular SPIONs [32]. However, when MSCs were incubated with ferumoxide in the absence of a facilitator the labeling of cells was negligible. Under these conditions incorporation was time dependent since after 24 hrs more Feridex incorporation occurred in the absence of facilitation than after 4 hrs of exposure.

In proliferation assays, we demonstrated that 4 hrs of incubation with FePLL did not alter MSC proliferative capacity. However, after 24 hrs of incubation with FePLL, we observed a reduction in proliferation rate that was not observed when MSCs were incubated with FeProt for either 4 or 24 hrs. The proliferation rate decrease observed in the FePLL 24 h group is dependent on the formation of FePLL complexes since incubation of the cells with PLL or Feridex alone did not affect proliferation rate. According to Kostura *et. al. *[39] the incorporation of FePLL complexes by MSCs affects their differentiation into chondrocytes. Incubation of PLL with Feridex can generate large FePLL complexes which can not be incorporated into endosomes and remain adhered to the cell membrane [31,40]. Recently it was demonstrated that labeling of MSCs with ferucarbotran, without an incorporation facilitator agent, inhibits chondrongenesis in a dose-dependent way. The authors suggest that surface binding of ferucarbotran SPIONs could inhibit surface-linked cell-cell interactions. This does not appear to happen when the MSCs are exposed to ferucarbotran associated with protamine because the compound can facilitate transport of the SPIONs into the intracellular compartment [47]. Our results show that the protocol using FeProt is superior to that using FePLL due to the toxicity observed when MSCs were cultivated with FePLL for 24 hrs.

It was suggested that relatively high concentrations of protamine (e.g., 5-6 μg/mL) form large extracellular complexes that are not incorporated by the cells but remain permanently attached to the cell membrane. Recently, some authors described an optimized protocol for cell labeling using lower concentrations of protamine and higher concentrations of Feridex than used in their previous studies. Formation of extracellular aggregates was not observed using this new protocol [32]. However, using the new optimized protocol, the authors did not test whether higher concentrations of protamine could induce the formation of extracellular complexes. In our study we propose an optimized protocol using a low concentration of Feridex and a higher concentration of protamine (25:5 μg/mL Fe:Prot). Using our protocol, extracellular aggregates attached to the MSC membrane were not observed by electron microscopy (unpublished data in collaboration with members of our laboratory, Louise Moraes and Wagner Monteiro Cintra).

Since protamine is clinically approved and does not alter proliferation rate, we performed a more extensive investigation evaluating FeProt complexes as candidates to label MSCs. We chose the protocol using a short-term (4 hrs) exposure to FeProt because this is more suitable for clinical use. When we monitored the cells, injected in the mouse leg muscles, by *in vivo *MRI, there were no apparent differences in the hypointense dark spots resulting from the MSCs incubated for short or long time periods with FeProt. Moreover, we could detect even a small number of the 4 hr FeProt labeled cells in the *in vitro *assays.

Other incorporation facilitators, besides protamine and PLL, have been used for cell labeling, such as FuGENE [13], Superfect and Lipofectamine [25] and some authors have not used any incorporation facilitator for cell labeling [48,49]. The primary advantage of the incubation labeling method is its simplicity. The primary disadvantage is the prolonged incubation time required [50]. Thus other approaches to induce labeling of freshly isolated cells such as magnetoelectroporation [51,52] and magnetosonoporation [53] appear to be better when the culture system must be avoided. Both techniques induce reversible eletromechanical permeability changes in the cell membranes, thereby facilitating the diffusion of MRI contrast agents.

Most authors have analyzed the effect of SPIONs on cell proliferation and viability by MTT (3-[4,5-dimethylthia-zol-2-yl]-2,5-diphenyl tetrazolinum bromide) assays [30,31,49]. However, it has been shown that the MTT test is unsuitable for measuring either cell growth or proliferation [54-56]. In the present study, we analyzed MSC proliferation based on detection of the Ki67 protein and viability by live/dead assays for up to 7 days after initial exposure to SPIONs. We did not observe alterations in MSC proliferation rate at 3 or 7 days after the initial 4 hr exposure to FeProt when compared with the respective controls. In addition, we analyzed the viability of MSCs after exposure to FeProt complexes. The viability was maintained after 4 or 24 hrs of incubation and at 3 or 7 days after initial 4 hr incubation with FeProt. Our results demonstrate that the number of cells labeled with Feridex is maintained after 7 days. However, after 21 days the number of labeled cells decreased by more than 65%, mainly due to cellular proliferation because when we cultured the labeled MSCs at confluence (FeProt 21d group) or pre-treated the cells with mitomycin C (FeProt MitC group), we observed only small decreases of 32.8% and 19.4% in the Feridex-labeled cell number, respectively. Moreover, even the small decreases observed in FeProt 21d and FeProt MitC groups were probably due to ongoing proliferation, since measurements of the group pre-treated with mitomycin C showed a sustained proliferation rate of 6.9% (data not shown). We believe that it is very important that a significant fraction of the cells retain the label in order to accurately report the distribution of the MSC population.

Although labeling of exogenous cells has been extensively used for tracking cells after transplantation, it is important to emphasize that endogenous cell labeling is essential for understanding the alterations of migratory activities in normal and injured organs and for the development of new therapies. Micrometer-sized superparamagnetic iron oxide particles (MPIOs) have been widely used for endogenous tracking of neural stem/progenitor cell migration from subventricular zone (SVZ) but are not yet clinically approved. Efficient endogenous labeling can be achieved using a large amount of MPIOs without an incorporation facilitator [19-21,57]. However, quantitative analysis of bromodeoxyuridine revealed altered proliferation in the SVZ and neural progenitor cells after *in situ *injection of MPIOs. When a small number of MPIOs was associated with PLL, labeling was more successful, and the proliferation in the SVZ was only marginally affected [21]. In our work we have focused on labeling of exogenous cells, but the knowledge about safe and efficient labeling is expected to be applicable to endogenous labeling as well.

Besides ferumoxides, ferucarbotran and gadolinium have also been used as clinical contrast agents for MRI and to label and track transplanted cells. Ferucarbotran (Resovist, Bayer Schering Pharma AG, Berlin, Germany) is a SPION coated with carboxydextran while ferumoxide (Feridex) is coated with dextran. It was suggested that the additional carboxyl groups associated with ferucarbotran might lead to a higher affinity to the cell membrane so that cells could be labeled with it without need of an incorporation facilitator [48,49]. However, recent work showed that a higher percentage of labeled cells, a higher amount of intracellular iron and lower amount of extracellular iron aggregates were reached using FeProt complexes when compared to Resovist without incorporation facilitator [33]. Moreover, in a study on human MSC (hMSC), there was no difference in the total iron content (pg/cell) among cells incubated with Feridex or Resovist when both were added together with PLL; both Feridex and Resovist incorporation was superior to a third type of nanoparticle [monocrystalline iron oxide (MION), an ultrasmall superparamagnetic iron oxide] when added with PLL [58]. Cell viability and proliferation were not altered in any condition. However, the levels of Oct-4 mRNA increased in labeled hMSC at day 1 but not at day 7 and a subpopulation of hMSCs expressing CD45 was observed after 7 days of culture.

Gadolinium nanoparticles are also clinically approved as contrast agents. Although gadolinium-labeled stem cells have been reported to be efficiently tracked by MRI [18,59], a significant increase in reactive oxygen species was observed at all time points (from 2 to 24 hrs) after cell labeling and a significant decrease in the proliferation rate was observed after 24 hours [60]. In addition, gadolinium-labeling can affect proteoglycan synthesis, cell proliferation and apoptosis of chondrocytes in a dose-dependent manner [61]. For these reasons, ferumoxides appear currently to be the preferred material for cell labeling and tracking.

For clinical protocol approval, it is essential to maintain the differentiation capacity of MSCs after exposure to FeProt complexes. The 4 hr Feridex labeled cells were induced to differentiate into adipocytes, osteocytes or chondrocytes and we observed that the differentiation capacity was unaffected by FeProt incorporation.

## 5. Conclusion

In summary, in this study we demonstrate that FePLL complexes affect cell proliferation after 24 hr exposure. However, the protocol using FeProt complexes does not affect the proliferative capacity and cellular viability for up to 7 days after incorporation. In addition, the differentiation potential of labeled MSCs is not affected. Furthermore, the protocol using FeProt complexes can be applied to patients, since both ferumoxides and protamine are approved for human use and our results show that this protocol is efficient to track cells by MRI.

## Competing interests

The authors declare that they have no competing interests.

## Authors' contributions

J conceived of the study, participated in the design, collection and assembly of data, performed the statistical analysis, interpretation and drafted the manuscript. ALMT, HMPN and JAP assisted with collection and assembly of data and performed the statistical analysis. LAJ collected data and helped draft the manuscript. ELG conceived of the study, participated in its design and coordinated its execution. DCS, ACCC and RMO conceived of the study, participated in its design and coordination and drafted the manuscript. All authors read and approved the final manuscript.
