# Correction: optimized labeling of bone marrow mesenchymal cells with superparamagnetic iron oxide nanoparticles and *in vivo *visualization by magnetic resonance imaging

**DOI:** 10.1186/1477-3155-9-12

**Published:** 2011-04-06

**Authors:** Jasmin, Ana M Luiza Torres, Henrique MP Nunes, Juliana A Passipieri, Linda A Jelicks, Emerson L Gasparetto, David C Spray, Antonio C Campos de Carvalho, Rosalia Mendez-Otero

**Affiliations:** 1 Instituto de Biofísica Carlos Chagas Filho, Universidade Federal do Rio de Janeiro, Rio de Janeiro, Brazil; 2 Dept. of Neuroscience, Albert Einstein College of Medicine, Bronx, NY, USA; 3 Dept. of Physiology and Biophysics, Albert Einstein College of Medicine, Bronx, NY, USA; 4 Hospital Universitário Clementino Fraga Filho, Universidade Federal do Rio de Janeiro, Rio de Janeiro, Brazil

## 

The methods of the published manuscript [1] state that an incorrect concentration of Feridex was used (25 ug/ml). Instead, the methods should state that a concentration of 50 ug/ml Feridex was used.
